# Prevalences of cardiometabolic risk and lifestyle factors in young parents: evidence from a German birth cohort study

**DOI:** 10.1186/s12872-022-02915-z

**Published:** 2022-11-07

**Authors:** Jana Brunner, Sara Fill Malfertheiner, Susanne Brandstetter, Birgit Seelbach-Göbel, Christian Apfelbacher, Michael Melter, Michael Kabesch, Andrea Baessler, KUNO-Kids Study Group, Petra Arndt, Mark Berneburg, Stephan Böse-O’Reilly, Romuald Brunner, Wolfgang Buchalla, André Franke, Sebastian Häusler, Iris Heid, Caroline Herr, Wolfgang Högler, Sebastian Kerzel, Michael Koller, Michael Leitzmann, David Rothfuß, Wolfgang Rösch, Bianca Schaub, Bernhard H. F. Weber, Stephan Weidinger, Sven Wellmann

**Affiliations:** 1 University Department of Obstetrics and Gynecology, Hospital St. Hedwig of the Order of St. John, University Medical Center Regensburg, Regensburg, Germany; 2 Member of the Research and Development Campus Regensburg (WECARE) at the Clinic St. Hedwig, Regensburg, Germany; 3 University Children’s Hospital Regensburg (KUNO-Clinics), University of Regensburg, Clinic St. Hedwig, Regensburg, Germany; 4 Institute of Social Medicine and Health Systems Research (ISMHSR), Otto von Guericke University Magdeburg, Magdeburg, Germany; 5 Clinic of Internal Medicine II, University Hospital of Regensburg, Franz-Josef-Strauß-Allee 11, 93053 Regensburg, Germany

**Keywords:** Cardiometabolic health, Birth cohort study, Health behavior, Lifestyle

## Abstract

**Background:**

Studies show that parents significantly impact their children’s health through their cardiometabolic risk profile and health behavior. There is only little information about the prevalence of cardiometabolic risk factors and lifestyle factors among new parents yet. The aims of this study are therefore to evaluate the prevalences of cardiometabolic risk factors in parents of infants in Germany and to examine their lifestyle and health behavior.

**Methods:**

In the KUNO-Kids health study, an ongoing birth cohort, parents (*n* = 930 mothers and 769 fathers) were asked about cardiometabolic risk factors (obesity/hypertension/type 2 diabetes mellitus) and lifestyle factors (dietary/sports/smoking habits/alcohol consumption) during the first year after the birth of their children via questionnaires. Chi-square as well as fisher exact tests were conducted to analyse associations between lifestyle factors and cardiometabolic risk factors.

**Results:**

34.2% of mothers and 58.5% of fathers were overweight or obese. In 11.8% of the families, at least one parent suffered from hypertension, in 2.4% from type 2 diabetes mellitus. One year after delivery, 8.5% of mothers were smoking, 6.9% showed a risky alcohol consumption (> 10 g/d). 16.0% of fathers were smoking 4 weeks after childbirth, 10.7% showed risky alcohol consumption (> 20 g/d). 21.6% of mothers carried out sports activity for more than 2 h a week then. Parental hypertension was linked to a higher prevalence of risky alcohol consumption, obesity to a lower prevalence of daily fruits consumption.

**Conclusions:**

Cardiometabolic risk factors were widespread among new parents with obesity and overweight having the highest prevalences. A considerable number of parents also practiced an unhealthy lifestyle showing that there is potential for improvement to promote the healthy development of their children.

**Supplementary Information:**

The online version contains supplementary material available at 10.1186/s12872-022-02915-z.

## Background

Cardiovascular disease and its underlying lifestyle and metabolic risk factors are responsible for about 31% of deaths worldwide [1].

Studies on cardiovascular risk factor burden usually focus on general or older populations and often exclude pregnant women when addressing younger populations. However, complications during pregnancy such as preeclampsia and gestational diabetes also predict cardiovascular morbidity and mortality [2, 3].

Pregnancy imposes some distinct physiologic changes upon the cardiovascular and metabolic systems that could make women vulnerable to hypertensive disorders of pregnancy and gestational diabetes, particularly when underlying pre-pregnant risk factors such as obesity, sedentary lifestyle with little physical activity and unhealthy nutrition, are present [4, 5].

Moreover, it is well known that the cardiometabolic risk is often transmitted into the next generation. It is not only clear that most if not all components of the metabolic syndrome aggregate within families, but maternal as well as paternal overweight and cardiovascular disease are also predictors of childhood adiposity [6–8]. The basis for some of these effects might be set at a very early point in time as the concepts of fetal and early life programming suggest that adverse conditions like hyperinsulinism in critical early periods of life (pre- and postnatal) could alter the metabolism of children having a lasting effect on their health and on the risk of obesity, diabetes, and cardiovascular diseases in later life [8, 9]. Although much of the agglomeration of cardiovascular risk factors within families has a genetic basis, environmental influences of a shared household contribute to a transmission of cardiometabolic risk from parents to their offspring as well [10].

Exemplarily, exposure to parental smoking does not only have a tremendous influence on the risk of lower respiratory infections in infants but is also associated with higher exercise systolic blood pressure and atherosclerotic vascular disease in the offspring [11–13]. Furthermore, children of current and former smokers are more likely to start smoking themselves [14]. Familial resemblance has also been observed for physical activity, dietary patterns and alcohol consumption [15–17].

Expecting and new parents might therefore be the right target for health prevention strategies aiming to prepare them for a healthy parenthood and child education with regard to healthy nutrition and lifestyle.

Indeed, while research shows that cardiometabolic risk factors and an unhealthy lifestyle are common among the general German population, there is only little information about the specific group of new parents among whom a considerable part showed a limited health literacy in previous studies [18–24].

The aims of this study are therefore to evaluate the prevalences of cardiometabolic risk factors like obesity, arterial hypertension and type 2 diabetes mellitus (T2DM) in parents of infants in Germany and to examine their lifestyle and health behavior (food habits, alcohol consumption, smoking habits and physical activity).

## Methods

### Data collection and measurements

This project is based on data from the KUNO-Kids health study, an ongoing birth cohort.

Since the start in 2015, all mothers planning to deliver their baby at the clinic St. Hedwig Regensburg and their partners have been invited to participate in the study. Exclusion criteria were mothers younger than 18 years of age or with insufficient German language skills limiting the understanding of the rationale of the study. Parents participated on a voluntary basis and provided written informed consent. The process was approved by the ethics board of the University of Regensburg in December 2014 (file number: 14–1010347).

**Fig. 1:**
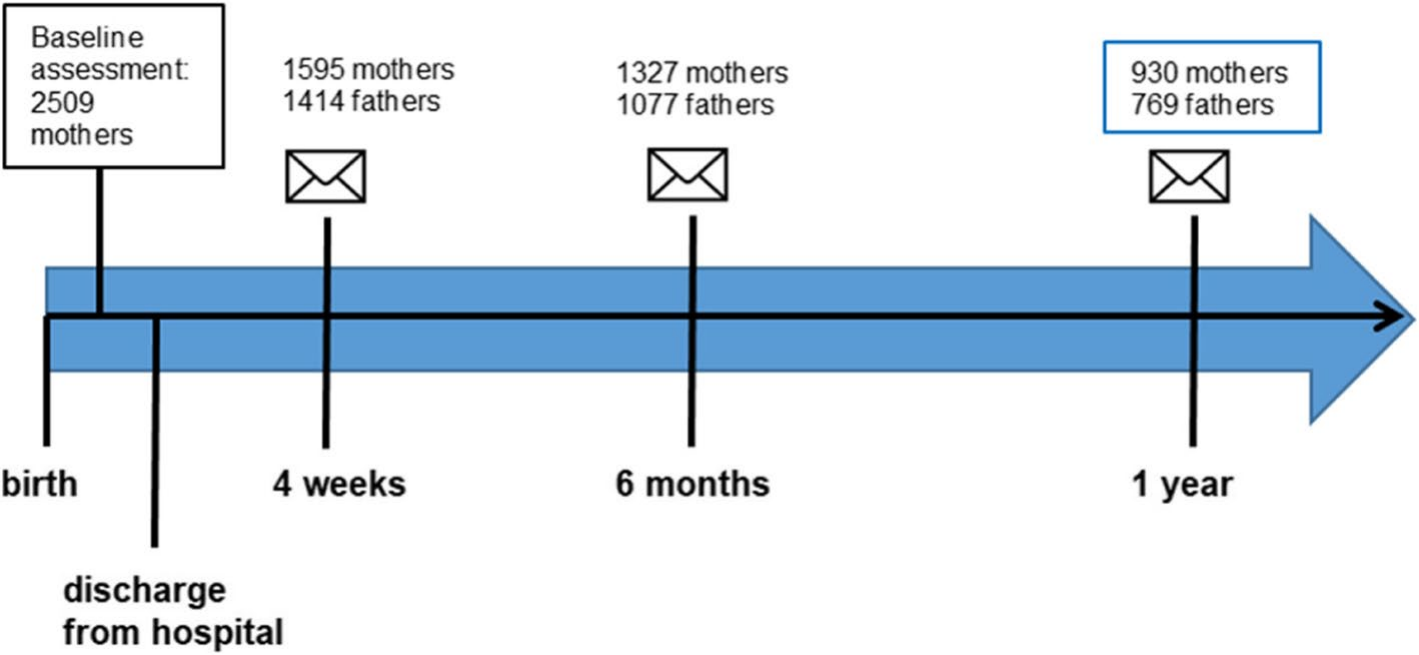
Participation throughout the course of the study

Information was gathered through an interview with the mothers after they gave birth and follow-up-questionnaires mailed to the parents after 4 weeks, 6 months and hereafter on every birthday of the child [25]. For this specific project, parents having participated in the study for at least 1 year were included. Included parents are shown in Fig. 1: Participation throughout the course of the study.

Parental heights and weights were collected through questionnaires (mothers before pregnancy and 1 year after child’s birth and fathers 4 weeks after child’s birth). Weight categories were defined according to WHO classifications (BMI < 18.5 ≙ underweight, BMI 18.5–25≙normal weight, BMI 25–30≙overweight, BMI ≥ 30≙obesity) [26]. One year after the child was born, information on parents suffering or ever having suffered from hypertension or T2DM (self-reported diagnosis) was gathered. Information on mothers having suffered from gestational diabetes mellitus (GDM) during the pregnancy with the study child was gathered in an interview shortly after birth.

Smoking habits were evaluated by asking whether parents had ever smoked more than 100 cigarettes in their life and whether they were currently (mothers 1 year after the child’s birth, fathers 4 weeks after the child’s birth) smoking. Participants indicating that they had never smoked more than 100 cigarettes in their life were defined as never-smokers. Participants who declared that they had smoked more than 100 cigarettes in their life but did not currently smoke were defined as ex-smokers and participants that answered that they currently smoked cigarettes were defined as smokers.

Quantities of recent alcohol consumption (amount of bottles (0.5 l) of light beer or beer, glasses of wine (0.25 l) or sparkling wine (0.1 l) and shots (0.02 l) consumed per week) were evaluated 1 year after the child’s birth for mothers and 4 weeks after the child’s birth for fathers. In previous studies, an alcohol consumption of more than 10–12 g/day for healthy women and 20–24 g/day for healthy men was described as risky alcohol consumption [27]. An alcohol consumption of more than 10 g/day for mothers and 20 g/day for fathers was therefore defined as risky alcohol consumption.

Maternal breastfeeding at the time of potential alcohol consumption and smoking was evaluated by asking whether their child was still fed breast milk.

Parental raw or cooked vegetables and fresh fruits consumption (never, once a month, several times a month, once a week, several times a week, almost daily or several times a day) as well as mothers engagement in sports (amount of hours per week) were evaluated 1 year after the child’s birth. A choosing of an “almost daily” or “several times a day” consumption of fresh fruits was counted as daily fruits consumption. A choosing of an “almost daily” or “several times a day” consumption of either raw vegetables and salads or cooked vegetables or a “several times a week” consumption of both raw vegetables and cooked vegetables was counted as daily vegetables consumption. Mothers’ ages were obtained through the St. Hedwig clinic network, fathers’ ages were collected in the 2-year follow-up questionnaire. Information on further socio-demographic characteristics of the study population (parental migration background, education and employment as well as maternal marital status) was collected through an interview shortly after birth and questionnaires 4 weeks and 1 year after birth.

### Statistics

Data was analysed using “SPSS” (version 25). Parental body mass indexes (BMI) and prevalences for parental overweight and obesity were calculated. For continuative analysis, a weighting factor taking into account the higher percentage of parents having graduated from grammar school in our study compared to the general population was applied [28].

Lifetime prevalences for parental hypertension and T2DM as well as prevalences for maternal GDM were calculated. Parental smoking habits were examined by calculating prevalences of never-smokers, ex-smokers and current smokers. Parental consumption of pure alcohol per day was calculated using common alcohol percentages (2.5% for light beer, 5% for beer, 11% for wine and sparkling wine and 38% for shots). Prevalences of parental risky alcohol consumption as well as daily fruits and vegetables consumption were calculated. Prevalences of maternal engagement in sports (none, < 2 h/week or > 2 h/week) were evaluated. 95% confidence intervals were calculated by bootstrap resampling.

Chi-square as well as fisher exact tests were conducted in order to analyse associations between lifestyle factors and cardiometabolic risk factors.

## Results

### Study population

Two thousand five hundred nine mothers agreed to participate in the KUNO-Kids study and completed the basic assessment. One thousand nine hundred thirty-six families have reached the 1-year follow-up and for this analysis a total of 930 mothers and 769 fathers could be included. Data on all variables analysed in this project was available for 599 mothers and 614 fathers. In this research project, the average age of mothers 1 year after delivery was 33.6 (±4.1) years. More than 60% of the parents had obtained a degree from a grammar school. The vast majority of mothers and fathers were of German origin (also see Table 1: Socio-demographic characteristics of the study population). For sociodemographic characteristics of the drop-outs see: Additional file 1: Basic characteristics of participants dropped out before 1-year-questionnaire.

**Table 1:** Socio-demographic characteristics of the study population

	Mothers (*n* = 930)		Fathers (*n* = 769)	
**Age in years (**mean (SD))	33.6 (±4.1)		36.8 (±5.6) (n_total_ = 484)	
	n	**%**	n	**%**
**Education/school leaving certificate**				
**no certificate (yet)**	4	**0.4**	7	**0.9**
**certificate from secondary modern school 9 years**	62	**6.7**	120	**15.6**
**certificate from secondary modern school 10 years**	269	**28.9**	156	**20.3**
**certificate from grammar school**	595	**64.0**	486	**63.2**
**Migration background**	89	**9.6**	51	**6.6**
**Employed before birth**	854	**91.8**		
**Employed (1 year after birth)**	292 (n_total_ = 915)	**31.9**	720 (n_total_ = 740)	**97.3**
**Marital status**				
**married, living with husband**	795	**85.5**		
**married, living without husband**	2	**0.2**		
**unmarried, living together with partner**	104	**11.2**		
**unmarried, without partner**	2	**0.2**		
**single parent**	12	**1.3**		
**divorced**	6	**0.6**		
**widowed**	1	**0.1**		

### Evaluation of cardiometabolic risk factors in parents

In 17.8% (95%CI: 15.0–20.5%) of families, one parent was obese, in 4.0% (95%CI: 2.6–5.5%) of families, both parents suffered from obesity. 9.0% (95%CI: 7.2–10.8) of mothers had already been obese before pregnancy (also see Table 2: Parental distribution into BMI classifications).

**Table 2:** Parental distribution into BMI classifications

	Mothers (*n* = 893)				Mothers before pregnancy (*n* = 925)			Fathers (*n* = 750)			
	n	%	95%CI	%^a^	n	%	95%CI	n	%	95%CI	%^a^
**under weight** (BMI < 18,5)	43	**4.8**	3.5–6.3	**4.4**	35	**3.8**	2.5–5.0	1	**0.1**	0.0–0.4	**0.1**
**normal weight** (BMI 18,5 – 24,9)	545	**61.0**	57.7–64.3	**57.3**	644	**69.6**	66.6–72.7	310	**41.3**	37.7–44.9	**38.3**
**overweight** (BMI 25,0 – 29,9)	205	**23.0**	20.2–25.7	**22.3**	163	**17.6**	15.3–20.1	327	**43.6**	39.9–47.1	**44.8**
**obesity** (BMI ≥ 30)	100	**11.2**	9.1–13.5	**16.1**	83	**9.0**	7.2–10.8	112	**14.9**	12.5–17.6	**16.8**

^a^weighting factor applied

4.7% (95%CI: 3.2–6.2%) of mothers and 7.4% (95%CI: 5.7–9.4%) of fathers had ever suffered from arterial hypertension. 2.2% (95%CI: 1.3–3.3%) of mothers and 0.1% (95%CI: 0.0–0.4%) of fathers had ever suffered from T2DM. 14.4% (95%CI: 12.0–16.8%) of mothers had suffered from GDM.

### Evaluation of lifestyle factors

In 14.7% (95%CI: 11.9–17.6%) of families, one parent was smoking and in 4.9% (95%CI: 3.4–6.7%) of families, both parents were smokers. In 14.6% (95%CI: 12.0–17.4%) of families, one parent had been engaging in risky alcohol consumption, in 2.3% (95%CI: 1.2–3.6%) of families, both parents had done so.

In 29.7% (95%CI: 26.3–33.2%) of families, no parent consumed fruits on a daily basis, in 25.1% (95%CI: 21.7–28.3%) of families, both parents did so. No parental daily vegetable consumption was found in 39.7% (95%CI: 35.8–43.2%) of families whereas in 24.6% (95%CI: 21.3–27.8%) of families, both parents showed daily vegetable consumption (also see Table 3: Lifestyle factors among parents during their child’s first year, Additional file 2:obesity and lifestyle factors in families as well as Additional file 3: Maternal alcohol and smoking habits by breastfeeding status).

**Table 3:** Lifestyle factors among parents during their child’s first year

	Mothers				Fathers			
**Alcohol consumption**	n	**%** (n_total_ = 886)	95%CI	**%**^a^	n	**%** (n_total_ = 735)	95%CI	**%**^a^
**none**	433	**48.9**	45.4–52.1	**51.4**	138	**18.8**	16.0–21.8	**20.4**
**moderate**	392	**44.2**	41.0–47.6	**42.4**	518	**70.5**	67.1–73.6	**68.4**
**risky**	61	**6.9**	5.3–8.6	**6.2**	79	**10.7**	8.4–13.2	**11.2**
**Dietary**	n	**%** (n_total_ = 899)	95%CI	**%**^a^	n	**%** (n_total_ = 714)	95%CI	**%**^a^
**daily fruits consumption**	563	**62.6**	59.3–65.8	**61.2**	233	**32.6**	29.2–36.2	**30.8**
**daily vegetables consumption**	447	**49.7**	46.5–52.9	**47.7**	242	**33.9**	30.5–37.4	**31.5**
**Smoking habits**	n	**%** (n_total_ = 845)	95%CI	**%**^a^	n	**%** (n_total_ = 742)	95%CI	**%**^a^
**never-smokers**	482	**57.0**	53.6–60.1	**54.4**	346	**46.6**	42.9–50.2	**42.8**
**ex-smokers**	291	**34.4**	31.3–37.8	**36.3**	277	**37.3**	34.0–41.0	**37.9**
**current smokers**	72	**8.5**	6.8–10.5	**9.3**	119	**16.0**	13.3–19.0	**19.3**

^a^weighting factor applied

31.5% (95%CI: 28.5–34.5%) of mothers did not engage in any sports. 21.6% (95%CI: 19.2–24.2%) of mothers did sports for more than 2 h per week.

### Associations between lifestyle factors and cardiometabolic risk factors

In the following, only significant results are listed, for all analysed relations, see Tables 4 and 5: Cardiometabolic risk factors stratified for lifestyle variables for mothers/fathers. Parents suffering from arterial hypertension were significantly more likely to consume risky amounts of alcohol (mothers: 17.9% vs. 6.3%, *p* = 0.036; fathers: 25.5% vs. 8.6%, *p* = 0.001). Obesity was linked to a lower prevalence of daily fruits consumption (mothers: 50.7% vs. 65.4%; fathers: 24.4% vs. 33.4%). This association was only statistically significant for mothers though (*p* = 0.014). Obese mothers were significantly less likely to engage in sports for at least 2 h per week (13.7% vs. 24.9%, *p* = 0.034). Never-smokers among mothers were significantly less likely to suffer from GDM (11.0% vs. 18.2%, *p* = 0.012).

**Table 4:** Cardiometabolic risk factors stratified for lifestyle variables for mothers

Mothers (***n*** = 599)															
	Obesity					Arterial hypertension					Type 2 diabetes mellitus				
	yes		no			yes		no			yes		no		
**Smoking**	n	%	n	%	p^a^	n	%	n	%	p^a^	n	%	n	%	p^a^
never	36	49.3	310	58.9	0.119	13	46.4	333	58.3	0.214	6	42.9	340	58.1	0.253
formerly or currently	37	50.7	216	41.1		15	53.6	238	41.7		8	57.1	245	41.9	
**Fruits consumption**	n	%	n	%	p^a^	n	%	n	%	p^a^	n	%	n	%	p^a^
daily	37	50.7	344	65.4	**0.014**	19	67.9	362	63.4	0.632	6	42.9	375	64.1	0.103
not daily	36	49.3	182	34.6		9	32.1	209	36.6		8	57.1	210	35.9	
**Vegetables consumption**	n	%	n	%	p^a^	n	%	n	%	p^a^	n	%	n	%	p^a^
daily	33	45.2	271	51.5	0.312	15	53.6	289	50.6	0.760	7	50.0	297	50.8	0.955
not daily	40	54.8	255	48.5		13	46.4	282	49.4		7	50.0	288	49.2	
**Alcohol consumption**	n	%	n	%	p^b^	n	%	n	%	p^b^	n	%	n	%	p^b^
none or moderate	71	97.3	487	92.6	0.212	23	82.1	535	93.7	**0.036**	12	85.7	546	93.3	0.248
risky	2	2.7	39	7.4		5	17.9	36	6.3		2	14.3	39	6.7	
**Sports**	n	%	n	%	p^a^	n	%	n	%	p^a^	n	%	n	%	p^b^
> 2 hours/week	10	13.7	131	24.9	**0.034**	4	14.3	137	24.0	0.237	4	28.6	137	23.4	0.749
< 2 hours/week	63	86.3	395	75.1		24	85.7	434	76.0		10	71.4	448	76.6	

^a^chi-square-test conducted

^b^ fisher-exact-test conducted

**Table 5:** Cardiometabolic risk factors stratified for lifestyle variables for fathers

Fathers (***n*** = 614)^a^										
	Obesity					Arterial hypertension				
	yes		no			yes		no		
**Smoking**	n	%	n	%	p^b^	n	%	n	%	p^b^
never	36	40.0	256	48.9	0.120	23	48.9	269	47.4	0.844
formerly or currently	54	60.0	268	51.1		24	51.1	298	52.6	
**Fruits consumption**	n	%	n	%	p^b^	n	%	n	%	p^b^
daily	22	24.4	175	33.4	0.093	18	38.3	179	31.6	0.342
not daily	68	75.6	349	66.6		29	61.7	388	68.4	
**Vegetables consumption**	n	%	n	%	p^b^	n	%	n	%	p^b^
daily	24	26.7	181	34.5	0.143	19	40.4	186	32.8	0.287
not daily	66	73.3	343	65.5		28	59.6	381	67.2	
**Alcohol consumption**	n	%	n	%	p^b^	n	%	n	%	p^c^
none or moderate	77	85.6	476	90.8	0.122	35	74.5	518	91.4	**0.001**
risky	13	14.4	48	9.2		12	25.5	49	8.6	

^a^ type 2 diabetes mellitus was not stratified for lifestyle variables for fathers due to n(T2DM)_fathers_ = 1

^b^ chi-square-test conducted

^c^ fisher-exact-test conducted

## Discussion

We demonstrate that a considerable number of parents report cardiometabolic risk factors and practice an unhealthy lifestyle with overweight and obesity constituting the most frequent health issue.

The prevalences of overweight and obesity among the parents in our study were slightly lower compared to other studies among the German general population of the same age [22]. After the application of a weighting factor taking into account the higher percentage of parents having graduated from grammar school in our study compared to the general population though, with 38% of mothers and 62% of fathers suffering from overweight and obesity our results were very similar to results from the general population of the same age. This goes along with previous findings that obesity is inversely correlated with socio-economic status, especially in women [29]. In 22% of the families, at least one parent suffered from obesity (also see Additional file 2: obesity and lifestyle factors in families). Almost every tenth mother suffered from obesity even before the pregnancy.

Parental obesity increases the risk of obesity in their children through genetic, biological and environmental influences and heightens the risk for cardiovascular disease in their offspring [7, 30, 31]. Furthermore, parental weight change was shown to be associated with weight change in their offspring and to alter the risk of their children to develop overweight [32, 33]. Maternal obesity during pregnancy is associated with GDM, preeclampsia and congenital anomalies within the fetus [34].

Given the widespread of overweight and obesity among parents found in our study, focusing on young parents’ cardiovascular health and especially parental obesity could therefore be an important factor to improve society’s overall and future health by benefitting both children and parents themselves.

According to perinatal statistics, the prevalence of gestational diabetes mellitus in Germany in 2016 was 5.4% which is clearly lower than the prevalence of 14% of mothers found in our study [35]. Melchior et al. already found a gestational diabetes prevalence of 13% in their study in 2017 though and figured that gestational diabetes prevalences were underestimated in Germany since their findings correlated with international estimates [36]. Our results support their considerations.

Comparable to the general German population, a considerable percentage of parents also reported the cardiometabolic risk factors arterial hypertension and T2DM [18, 37, 38]. Slight prevalence differences could be explained by the fact that previous studies did not distinguish between diabetes mellitus type 1 and 2 while we specifically asked about T2DM. Furthermore, we only conducted a survey, the estimated number of unreported cases might be higher given the known large number of undetected cases [39].

It is especially important to monitor and control maternal hypertension at childbearing age since during a possible subsequent pregnancy, a poorly-controlled blood pressure during the first trimester in pre-pregnancy hypertensive women could harm both the mother and the fetus increasing the risk for organ damage, low birth weight and pre-eclampsia [40].

Keeping in mind that especially for mothers, pregnancy may trigger the development of cardiovascular disease, a bigger focus on cardiovascular health post-partum and during the child’s early life could help to decelerate the progression of existing cardiovascular morbidity and prevent the development of further disease.

### Lifestyle factors among parents

With 9% of mothers and 16% of fathers currently smoking, the prevalences of current smokers among our parents were clearly lower than the prevalences found in previous studies among the general German population. This applies accordingly to the group of mothers in our study who were not breastfeeding anymore at this point of time (also see Additional file 3: Maternal alcohol and smoking habits by breastfeeding status) [23]. This suggests that smokers might have stopped doing so before or shortly after becoming a parent. Still, almost every fifth of our study children grew up in a household with at least one smoking parent (also see Additional file 2: obesity and lifestyle factors in families).

While the prevalences of risky alcohol consumption among the non-breastfeeding mothers and fathers in our study were somewhat similar to results from previous studies among the general German population, with 40% of mothers and 19% of fathers, the prevalence of never-drinkers among our parents was clearly higher [41]. Parents of an infant might have less time or possibilities to go out or meet with other adults only and therefore might have fewer occasions to drink alcohol. Similarly to previous findings, we found relations between arterial hypertension and risky alcohol consumption [42].

While daily fruits and vegetables consumption was more common among our parents than among the general population in previous studies, in almost 40% of families, no parent consumed fruits daily and in almost 30% of families, no parent consumed vegetables daily [19, 20]. Considering that the recommendation of the “Deutsche Gesellschaft für Ernährung” (dge) is not only to consume fruits and vegetables daily but to have five portions a day, there is still much need for improvement in order for the parents to be a good role model for their kids [43]. Furthermore, we found that obese parents were less likely to consume fruits on a daily basis, which goes along with previous studies [44].

Like in previous studies among the general German population, more than 30% of the mothers in our study did not engage in any sports [45]. This shows that there is still room for significant improvement also to help reduce the prevalence of obesity keeping in mind the relations found in our and previous studies [46]. According to WHO guidelines, adults aged 18 to 64 should do at least 150 minutes of moderate-intensity aerobic physical activity per week or an equivalent combination of moderate- and vigorous-intensity activity [47].

Keeping in mind the immense influence of parents on their children’s health, parent education programs may be employed (for example in birth preparation seminars or in the child’s early screening examinations) with a greater focus on the impact of parental obesity, cardiometabolic health and associated lifestyle factors on their offspring both pre- and postnatal to improve awareness of healthy parenting behaviours and activities, and promote healthy parent–child relationships.

### Strength and limitations of the study

The large study population and the data from both mothers and fathers enabled us to provide information in a broader context than most other cohort studies in a specific population sample of parents of infants. Nevertheless, in some analyses with small prevalences, the number of total cases is still very small indicating a limitation of the study.

As it is common in longitudinal cohort studies, there was a significant drop-out of parents throughout the course of the study. With the response rate being about 50% among the families having reached the 1-year follow-up, attrition bias might occur [25]. Furthermore, the drop-out among participants with a migration background was larger than among the participants of German origin which might lead to additional bias and therefore indicates another limitation of the study.

All information used in this study is self-reported by the parents. It might be prone to information bias like recall-bias or social desirability bias, especially for the lifestyle-related questions. Furthermore, due to a data collection only 2 years after delivery and a significant drop-out until that point of time, fathers’ ages are only known for 63% of the fathers. These fathers were 3.3 years older than the corresponding mothers on average which goes along with German birth statistics so we assumed the ages of the remaining fathers to be accordingly [48]. Information on mothers’ medical records was collected through a paper questionnaire given to the mothers shortly after birth. Apparently, it did not reach all mothers or was not sent back properly by all mothers. This explains the smaller basic population for some questions. Prevalences of certain risk factors are reported for different query periods for mothers and fathers. For example, paternal alcohol consumption and smoking habits were evaluated 4 weeks after birth. With mothers still being in childbed at this point of time and the majority of them still breast-feeding we thought it to be more reasonable to gather information on maternal smoking habits and alcohol consumption only 1 year after delivery. The different query periods may possibly have an influence on the prevalence of the risk factors in question.

## Conclusions

In summary, we found that comparable to the general population, a substantial number of young parents reported cardiometabolic risk factors, particularly overweight and obesity. A considerable number of parents also engaged in an unhealthy lifestyle which shows that there is still potential for improvement in order to improve society’s todays and future cardiometabolic health.

## Supplementary Information

**Additional file 1.** Basic characteristics of participants dropped out before 1-year-questionnaire.

**Additional file 2.** Obesity and lifestyle factors in families.

**Additional file 3.** Maternal alcohol and smoking habits by breastfeeding status.

## Data Availability

The datasets generated and/or analysed during the current study are not publicly available as they are part of the KUNO-Kids health study, an ongoing birth cohort with a still growing dataset that includes many more variables than the ones analysed in the current study. However, the datasets are available from the corresponding author on reasonable request.
