# Supplementary material for: Prevalences of cardiometabolic risk and lifestyle factors in young parents: evidence from a German birth cohort study
Source: BMC Cardiovasc Disord. 2022 Nov 7;22:469. doi: 10.1186/s12872-022-02915-z (PMC9641866; doi:10.1186/s12872-022-02915-z)
Supplement: Supplementary file 1 — Additional file 1. Basic characteristics of participants dropped out before 1-year-questionnaire. [file 12872_2022_2915_MOESM1_ESM.docx]

**Additional file 1** Basic characteristics of participants dropped out before 1-year-questionnaire

|  | **mothers** | | **fathers** |
| --- | --- | --- | --- |
|  | Participated at least in basic interview  (n=1579) | Participated at least until 4 weeks after delivery  (n=732) | Participated at least until 4 weeks after delivery (n=645) |
| **average age in years** | 33,0 | 33,3 |  |
| **education/school leaving certificate** |  |  |  |
| **no certificate (yet)** | 1.1 % | 0.1 % | 1,2 %  **0.9** |
| **certificate from secondary modern school 9 years** | 12.4 % | 7.9 % | 18.0 %  **15.6** |
| **certificate from secondary modern school 10 years** | 34.3 % | 36.2 % | 23.3 %  **20.3** |
| **certificate from grammar school** | 52.1 % | 55.7 % | 57.5 %  **63.2** |
| **migration background** | 18.0 % | 11.9 % | 10.2 %  **6.6** |
| **employed before birth** | 85.1% | 88.1% |  |
| **employed** |  |  | 96.9% |
| **marital status** |  |  |  |
| **married, living with husband** | 76.7% | 80.6% |  |
| **unmarried, living with partner** | 20.0% | 17.1% |  |
| **unmarried, without partner** | 1.7% | 1.0% |  |
| **divorced** | 1.5% | 1.2% |  |
| **widowed** | 0.1% | 0.1% |  |
