# Supplementary material for: Prevalences of cardiometabolic risk and lifestyle factors in young parents: evidence from a German birth cohort study
Source: BMC Cardiovasc Disord. 2022 Nov 7;22:469. doi: 10.1186/s12872-022-02915-z (PMC9641866; doi:10.1186/s12872-022-02915-z)
Supplement: Supplementary file 2 — Additional file 2. Obesity and lifestyle factors in families. [file 12872_2022_2915_MOESM2_ESM.docx]

**Additional file 2:obesity and lifestyle factors in families**

|  | **no parent** | | | **one parent** | | | **both parents** | | |
| --- | --- | --- | --- | --- | --- | --- | --- | --- | --- |
| **obesity** (from n_total_=703) | n | % | 95%CI | n | % | 95%CI | n | % | 95%CI |
|  | 550 | 78.2 | 75.3-81.3 | 125 | 17.8 | 15.0-20.5 | 28 | 4.0 | 2.6-5.5 |
| **current smokers** (from n_total_ =713) | n | % | 95%CI | n | % | 95%CI | n | % | 95%CI |
|  | 573 | 80.4 | 77.3-83.4 | 105 | 14.7 | 11.9-17.6 | 35 | 4.9 | 3.4-6.7 |
| **daily fruits consumption** (from n_total_=680) | n | % | 95%CI | n | % | 95%CI | n | % | 95%CI |
|  | 202 | 29.7 | 26.3-33.2 | 307 | 45.1 | 41.5-48.9 | 171 | 25.1 | 21.7-28.3 |
| **daily vegetables consumption** (from n_total_=680) | n | % | 95%CI | n | % | 95%CI | n | % | 95%CI |
|  | 270 | 39.7 | 35.8-43.2 | 243 | 35.7 | 32.4-39.3 | 167 | 24.6 | 21.3-27.8 |
| **risky alcohol consumption** (from n_total_=687) | n | % | 95%CI | n | % | 95%CI | n | % | 95%CI |
|  | 571 | 83.1 | 80.0-85.8 | 100 | 14.6 | 12.0-17.4 | 16 | 2.3 | 1.2-3.6 |
