# Supplementary material for: Prevalences of cardiometabolic risk and lifestyle factors in young parents: evidence from a German birth cohort study
Source: BMC Cardiovasc Disord. 2022 Nov 7;22:469. doi: 10.1186/s12872-022-02915-z (PMC9641866; doi:10.1186/s12872-022-02915-z)
Supplement: Supplementary file 3 — Additional file 3. Maternal alcohol and smoking habits by breastfeeding status. [file 12872_2022_2915_MOESM3_ESM.docx]

**Additional file 3** Maternal alcohol and smoking habits by breastfeeding status

|  |  | **still breastfeeding**  **at consumption period/point of time** | | | **not (anymore)breastfeeding**  **at consumption period/point of time** | | | |
| --- | --- | --- | --- | --- | --- | --- | --- | --- |
| **alcohol consumption** |  | n | **%**  (n_total_=438) | 95%CI | n | **%** (n_total_=392) | 95%CI | |
|  | **none** | 253 | **57.8** | 53.2-62.7 | 158 | **40.3** | 35.6-44.8 | |
|  | **moderate** | 170 | **38.8** | 33.7-43.3 | 195 | **49.7** | 45.0-55.0 | |
|  | **risky** | 15 | **3.4** | 1.9-5.3 | 39 | **9.9** | 7.0-13.3 | |
| **smoking habits** |  | n | **%** (n_total_=166) | 95%CI | n | **%** (n_total_=616) | 95%CI | |
|  | **never-smokers** | 108 | **65.1** | 57.3-72.2 | 338 | **54.9** | | 50.9-58.7 |
|  | **ex-smokers** | 55 | **33.1** | 26.0-40.6 | 218 | **35.4** | | 31.9-39.1 |
|  | **current smokers** | 3 | **1.8** | 0.0-4.0 | 60 | **9.7** | | 7.5-12.1 |
